# Efficacy of neuroendoscopic-assisted surgery in the management of symptomatic sacral perineural (Tarlov) cysts: a technical report

**DOI:** 10.3389/fsurg.2024.1307460

**Published:** 2024-02-29

**Authors:** Ying Jiang, Lan Dong, Yi-Qi Pan, Jun Zhao, Wen-Fang Li, Teng-Fei Zhang, Ting Zhang, Da-Wei Dai

**Affiliations:** ^1^ Cerebrovascular Diseases Center, Department of Neurosurgery, Renji Hospital, Shanghai, China; ^2^ Department of Emergency, Shanghai Chang Zheng Hospital, Shanghai, China; ^3^ Department of Otorhinolaryngology, Shanghai Jiao-Tong University School of Medicine Ruijin Hospital, Shanghai, China; ^4^ Department of Neurosurgery, Shanghai University of Medicine & Health Science Affiliated Zhoupu Hospital, Shanghai, China; ^5^ Department of Neurosurgery, Renji Hospital, Shanghai, China

**Keywords:** Tarlov cyst, neuro-endoscope, leakage point, fibrin, tamponade, laminectomy

## Abstract

**Introduction:**

The Tarlov cysts are pathological enlargements of the cerebrospinal fluid spaces between the endoneurium and perineurium, which can cause intolerable sciatic pain, motor impairment of lower limbs, and bladder/bowel dysfunction. Currently, the treatment results are unsatisfactory due to the low cure rates and extensive surgical trauma. Thus, there is an ongoing exploration of surgical techniques for Tarlov treatment. In the current study, we present a novel neuroendoscopic-assisted technique that combines the fenestration, leakage sealing, and tamponade of the Tarlov cyst.

**Methods:**

Between January 2020 and December 2021, a total of 32 Tarlov patients were enrolled and received neuroendoscopic-assisted surgery. Their pre- and post-surgical Visual Analogue Scale (VAS) scores, major complaints, and MR imaging were recorded for comparison.

**Results:**

27 of 32 patients (84.4%) patients demonstrated immediate pain relief as their VAS scores decreased from 5.6 ± 1.5 to 2.5 ± 1.1 (*p* < 0.01) on the first day after surgery. At the 3-month follow-up, the patients' average VAS score continued to decrease (1.94 ± 0.8). Meanwhile, saddle paresthesia, urinary incontinence, and constipation were relieved in 6 (50%), 4 (80%), and 5 (41.7%), respectively, according to patients self-report. No surgical-related complication was observed in any of the cases.

**Discussion:**

We conclude that neuroendoscopic-assisted surgery is an effective surgical method for symptomatic Tarlov cysts with minimized complications.

## 1 Introduction

The perineural cysts are also known as the Tarlov cysts since it was originally reported by Tarlov in 1938 as an incidental finding at autopsy (1). It is now known that the Tarlov cysts are pathological enlargements of cerebrospinal fluid (CSF) spaces between the endoneurium and perineurium (2), which are most commonly identified in the sacral spinal canal between the nerve root origin and the dorsal root ganglion. While the exact pathogenesis remains uncertain, trauma seems to be pre-deposited in most Tarlov cases (3–6). Other causes may include inflammation, congenital origin, degeneration, and genetic inheritance (4). These factors result in a ball-valve structure, allowing CSF to influx from the normal subarachnoid space via pulsatile and hydrodynamic forces while restricting CSF efflux, which promotes the perineural cysts to fill and expand in size (7).

The prevalence of Tarlov cysts was found to be 4.6%–9% in the overall population (5, 8). Despite the high prevalence, the Tarlov cysts are usually ignored during imaging exams because most patients are asymptomatic. Yet, there is a subset (approximately 1% or less) of Tarlov patients who tend to be symptomatic (8–10). The clinical presentations of symptomatic Tarlov cysts are related to S2, S3, or S4 compression, which leads to pain in the lower back, sacrococcygeal and perineal areas, somatic sensory disorder, functional impairment (motor weakness and claudication) of lower limbs, bladder and bowel dysfunction, and impotence (11–13). However the continuous research regarding the effect of various treatment options, there remains no consensus on the optimal therapy for symptomatic Tarlov cysts.

In the current study, we aimed to report a new surgical technique by using the neuro-endoscope to treat Tarlov cysts.

## 2 Material and methods

The current study is a single-center, non-blinded, prospective cohort study in a tertiary medical center**.** The research protocol was approved by the institutional ethics committee of Shanghai Chang Zheng Hospital (No.202009-N34). The authors asserted that all procedures contributing to this work comply with the ethical standards of the relevant national and institutional committees on human experimentation and with the Helsinki Declaration of 1975, as revised in 2008.

### 2.1 Surgical indication & patients' enrollment

Patients were recommended for surgery for the following indication: 1) patients presented Tarlov cyst related symptoms, and 2) the symptoms were not relieved due to medication failure, side effects, or complications.

Patients were recruited into this study if they fulfilled the following inclusion criteria: (1) >18 years old; (2) presentation of clinical signs and symptoms (including pain in the lower back, sacrococcygeal and perineal areas, somatic sensory disorder, functional impairment of lower limbs, bladder and bowel dysfunction, and impotence); (3) the Tarlov cysts diagnosis was confirmed on magnetic resonance imaging; (4) no presence of any contraindication of lumbar surgery; and (5) voluntary provision of written informed consent.

Individuals were excluded if they had multilevel lumbar disc disorder (>3), lumbar scoliosis, previous lumbar surgery, previous spine or pelvic fractures, vertebrate tumors or infectious diseases, and congenital spinopelvic anomalies. Overall, this study enrolled and reported the outcomes of the 32 patients who underwent surgery between January 2020 and December 2021.

### 2.2 Surgical procedure

All patients received perioperative management as previously indicated (14). The surgery was performed under general anesthesia by endotracheal intubation. The patient was in a prone position on a standard operating table. A 5- to 10-cm midline incision was performed right above the Tarlov cyst. The subcutaneous tissue was gradually opened sharply on the midline until the medial sacral crest was observed. A standard sacral laminectomy was performed. Care is given since the sacral nerve root fibers are usually running under the site of laminectomy. The thin transparent cyst wall membrane was widely fenestrated longitudinally with a micro-scissor, where no nerve fibers were detected. The 30-degree endoscope was inserted into the cyst via the stoma, which provided a panoramic surgical view, especially allowing for clear observation of the cyst roof. Emphasis was also placed on careful observation of the nerve root fibers to avoid any iatrogenic injury. Sufficient cyst CSF drainage was required to decompress the spinal subarachnoid space, which was crucial for identifying the leakage point of the cyst. After the leakage point was identified, a muscle graft was obtained below the subcutaneous opening and positioned within this communication. Fibrin glue was used to keep the muscle graft in place while further strengthening the sealing effect. The remnant cyst space was tamponed with autologous fat obtained from the buttock. Since the leakage point had already been sealed, no attempt was made to perform complete cyst tamponade as excessive filling will cause compression to the nerve root. The sacral laminoplasty was performed by using a set of titanium mini-plates to replace the native lamina if necessary. The skin was closed in its corresponding layers.

### 2.3 Study design

Two follow-ups are required. The first follow-up is performed on the first post-surgical date. The patients are asked to assess symptom relief regarding their primary discomfort before surgery. For those suffering from pain in the sacrococcygeal regions and/or lower extremities, the Visual Analogue Scale (VAS) is ordered. Since the patients have not fully recovered, a physical examination is not performed during this follow-up session. During the second follow-up (3rd month post-surgical date), patients are asked about their recovery, followed by a thorough physical examination, especially focusing on the sacral spinal cord nerve function. In addition, the VAS and MR imaging of the lumbosacral spine are ordered to understand their recovery objectively as well as to compare with their pre-surgical status.

### 2.4 Statistical analysis

The data was presented in the form of mean ± standard deviation (SD). GraphPad® Prism 8.0 (GraphPad Prism Software Inc, San Diego, CA, USA) was used for statistical analyses. Pairwise comparisons of the groups in terms of VAS were carried out using the paired *t*-test. A statistically significant difference was defined as *p* < 0.05.

## 3 Results

### 3.1 Overall pre- and post-surgery comparison

Twenty female and 12 male participants aged 45.7 ± 12.9 took part in this study. Prior to surgery, the majority of patients (31 cases, 96.9%) reported pain in the sacrococcygeal regions and/or lower extremities as their main complaint, with Visual Analogue Scale (VAS) scores averaging 5.6 + 1.5. Other reported complaints included saddle paresthesia (12 cases, 37.5%), urinary incontinence (5 cases, 15.6%), constipation (12 cases, 37.5%), and sexual dysfunction (1 case, 3.1%).

On the first day after the surgery, 27 patients (84%) reported significant pain relief, whose VAS score was significantly dropped as compared to their pre-surgical score (2.5 ± 1.1, *p* < 0.01). No surgical complication was observed in any of the patients. Fever was observed in 2 patients on the first post-surgical day. However, no infectious cause was identifiable. Since both patients presented excessive wound exudate, intravenous antibiotics and symptomatic treatment (amidopyrine & dexamethasone) were prescribed to prevent possible meningitis caused by either bacteria or fibrin. Patients' volume of wound exudate decreased significantly on the second post-surgical day, and their body temperature returned to normal after three days of administration.

At the 3-month follow-up, the average VAS score of the patients decreased further (1.94 ± 0.8), which was significantly lower than their first post-surgical follow-up score (*p* < 0.05). According to the patients' self-report, saddle paresthesia, urinary incontinence, and constipation were relieved in 6 (50%), 4 (80%), and 5 (41.7%) cases, respectively. Sexual dysfunction persisted in 1 patient. There was no recurrence of cysts observed on MR imaging in any of the patients.

### 3.2 Illustrative case

#### 3.2.1 Case 1

**Figure 1:**
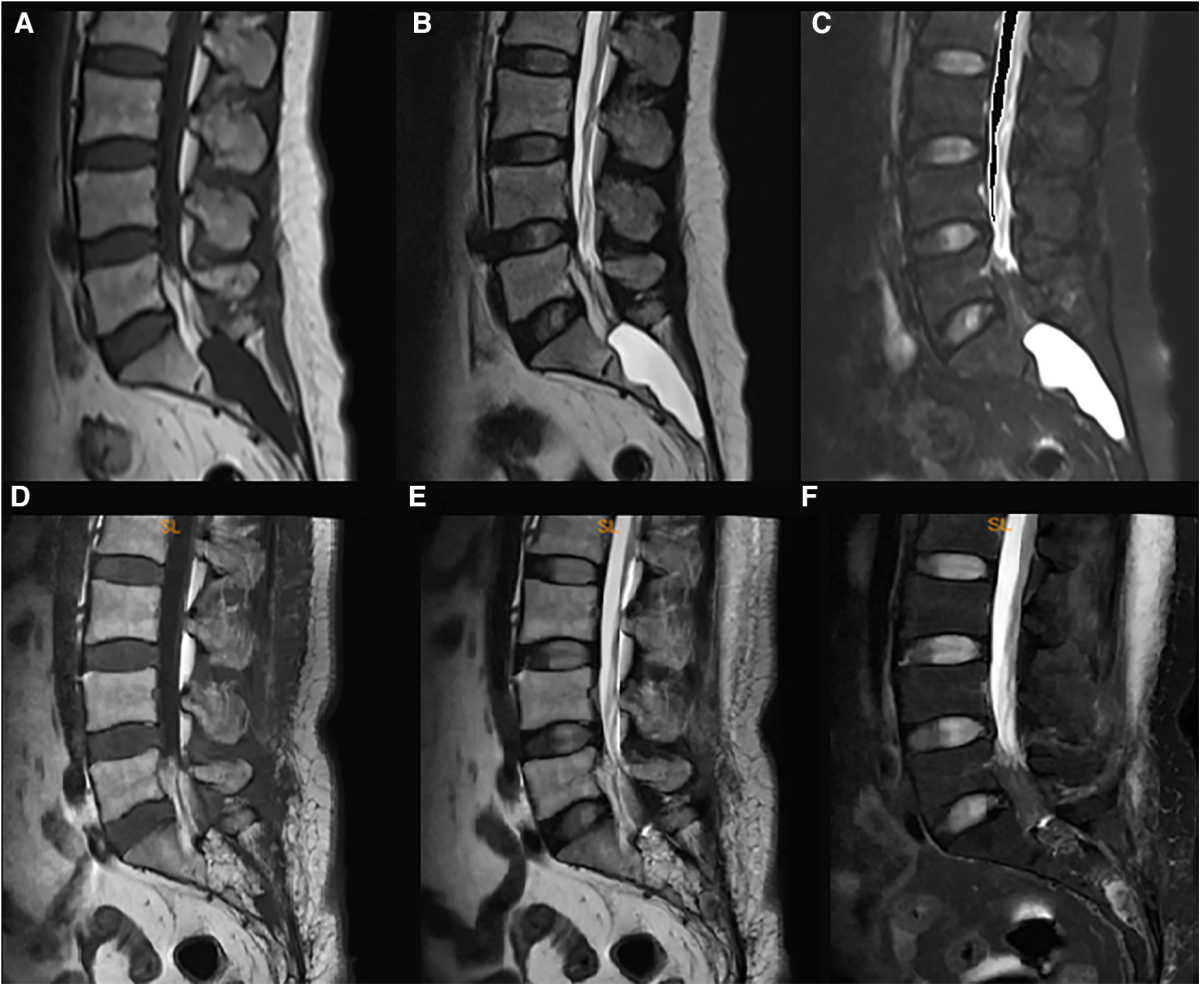
The pre- and post-surgical MR image of a symptomatic Tarlov cyst patient (case 1). Before surgery, the T1-weighted (**A**), T2-weighted (**B**), and fat suppression (**C**) sequences indicated that a Tarlov cyst filled with cerebrospinal fluid was in the sacral canal. After the surgery, the fat tissue occupied the original space of the Tarlov cyst, and no cerebrospinal fluid was detected in the sacral canal anymore (**D–F**), which indicated no recurrence of the Tarlov cyst.

We illustrated a 55-year-old female patient presented with a one-year history of pain in the sacrococcygeal region along with constipation. MR imaging of the lumbosacral spine demonstrated a 5.1 cm × 2.8 cm mass with homogenously bright signals on the T2-weighted sequence while dark signals on the fat-suppression sequence in the sacral canal (Figures 1A–C), which suggested the diagnosis of Tarlov cyst. The standard surgery as described was performed.

**Figure 2:**
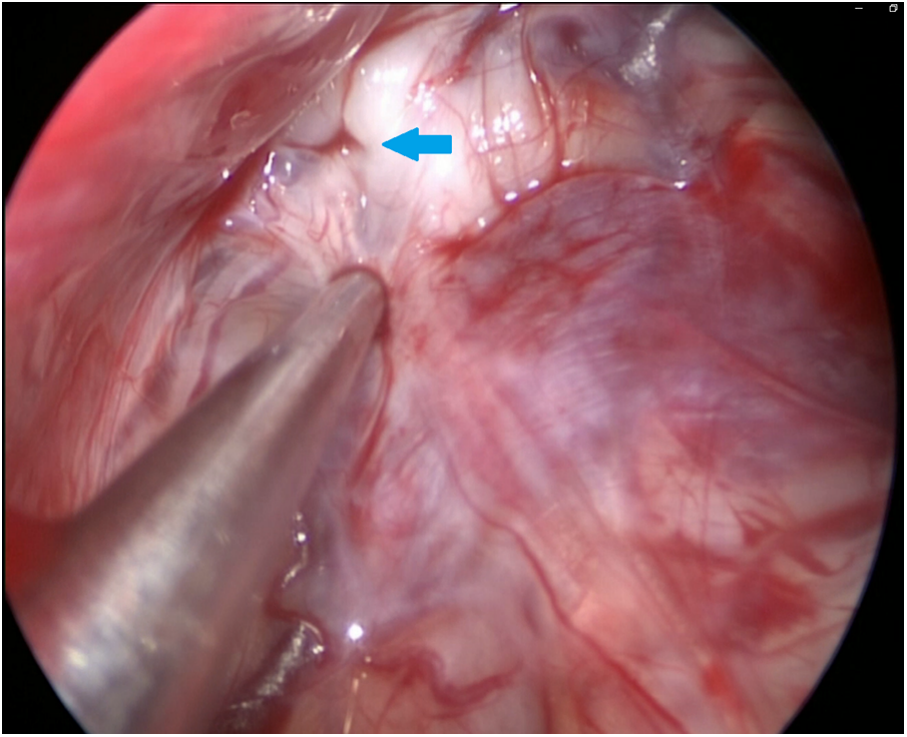
The 30-degree endoscope was inserted into the Tarlov cyst via the stoma and identified the communication on the roof of the cyst (as indicated by the arrow).

**Figure 3:**
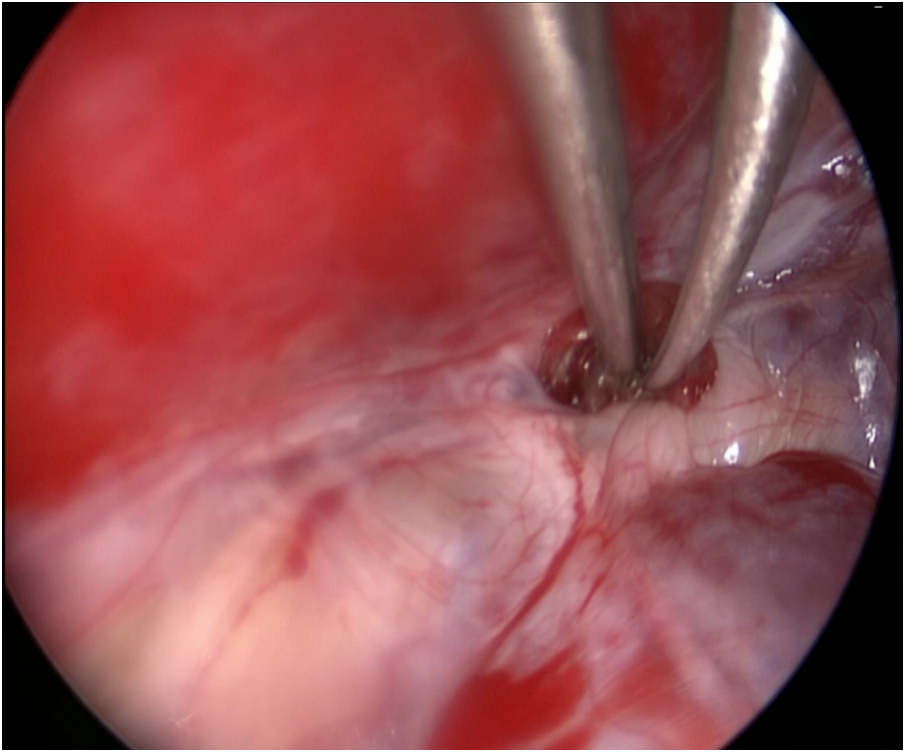
A muscle graft was obtained below the subcutaneous opening and positioned within the communication between the Tarlov cyst and subarachnoid space.

**Figure 4:**
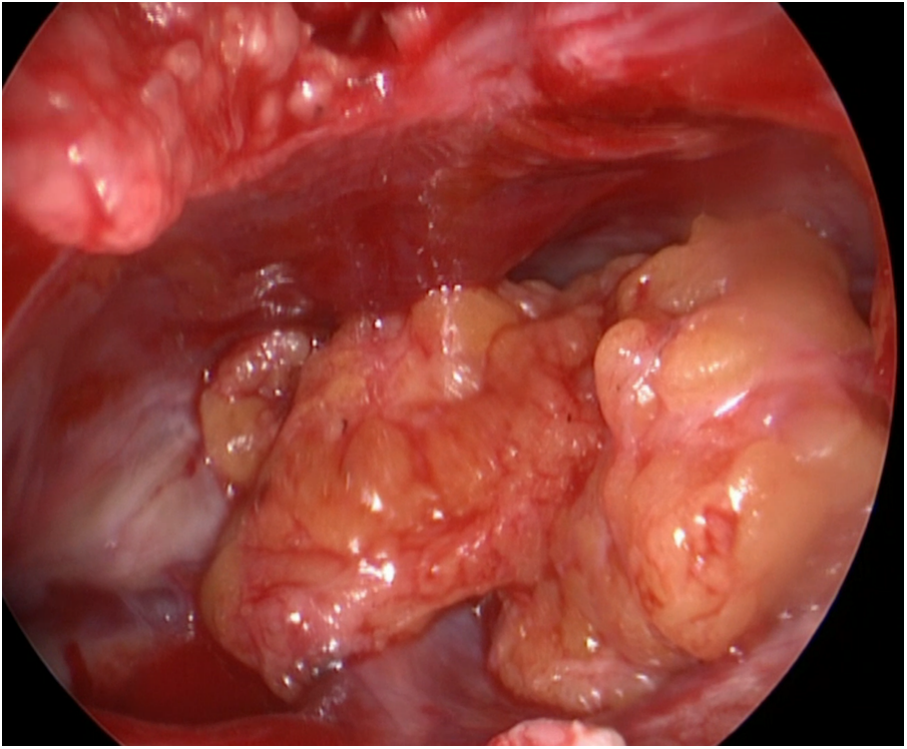
The remnant cyst space was tamponed with autologous fat obtained from the buttock.

During the surgery, the leakage site was identified at the roof of the Tarlov cyst (Figure 2), which was sealed by a muscle graft (Figure 3). The remnant cyst space was tamponed with autologous fat obtained from the buttock (Figure 4).

On the first post-surgical date, the patient reported apparent relief in pain relief. At the 3rd-month follow-up, the patient reported total pain relief with an apparent resolution of constipation. Follow-up MRI did not demonstrate evidence of cyst recurrence (Figures 1D–F).

#### 3.2.2 Case 2

**Figure 5:**
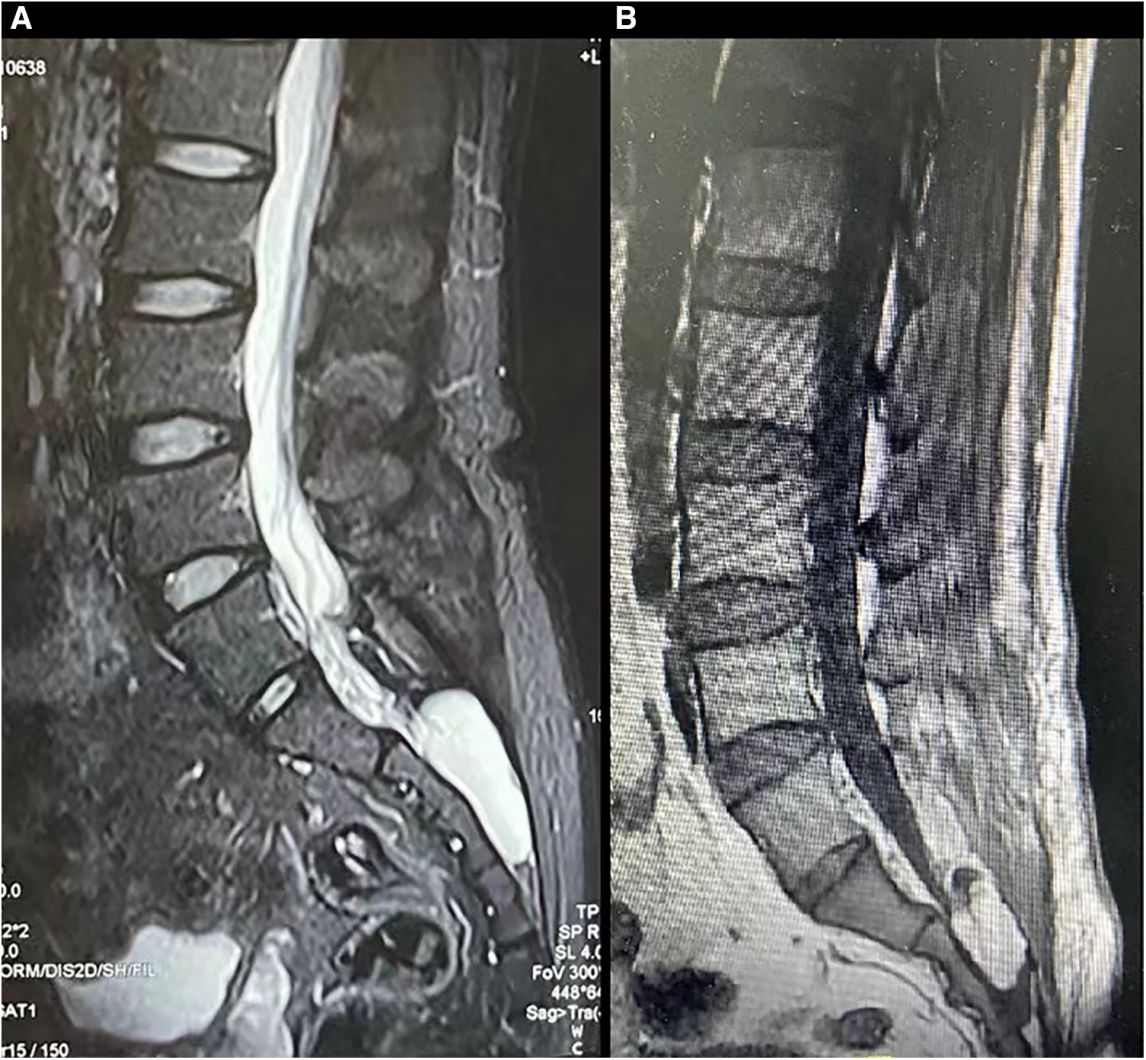
The MR images of a patient with a symptomatic Tarlov cyst before and after surgery were presented (case 2). Before surgery, T2-weighted sequences (**A**) revealed the presence of a Tarlov cyst filled with cerebrospinal fluid in the sacral canal. Following surgery, the original space occupied by the Tarlov cyst was replaced by fat tissue (**B**).

A male patient, aged 48, visited our clinics due to a one-year history of back pain, erectile dysfunction, and urinary incontinence. A large Tarlov cyst (5.1*2.3 cm) was detected at the S2-3 level on the lumbosacral MR (Figure 5A). The patient received the surgery as described.

**Figure 6:**
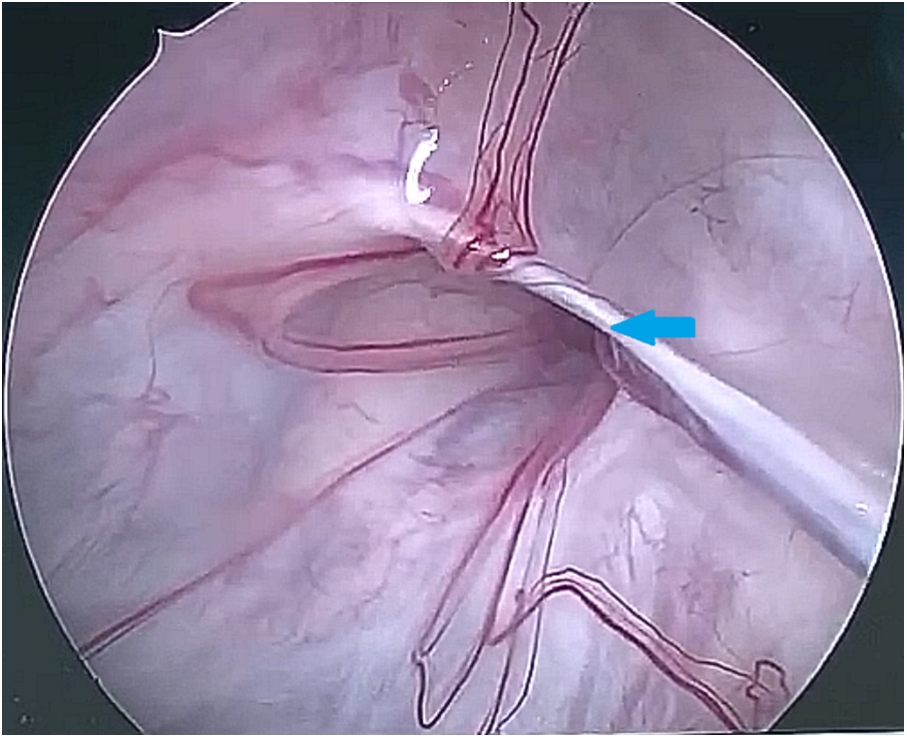
The 30-degree endoscope was inserted through the stoma into the Tarlov cyst, identifying the communication on the cyst roof (as indicated by the arrow).

**Figure 7:**
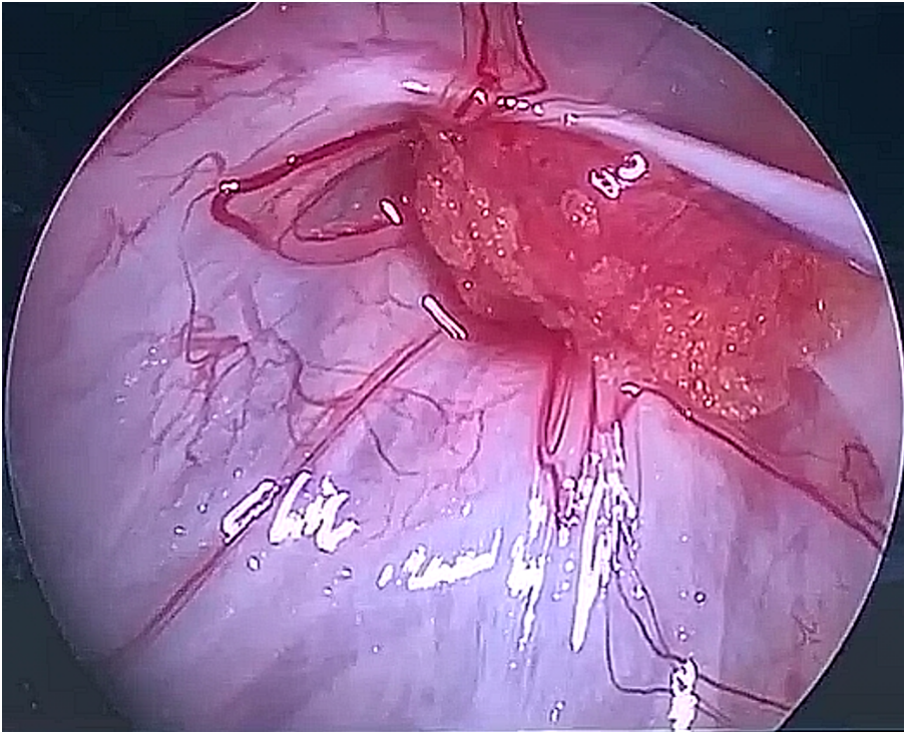
A muscle graft was used to seal the communication between the Tarlov cyst and subarachnoid space.

**Figure 8:**
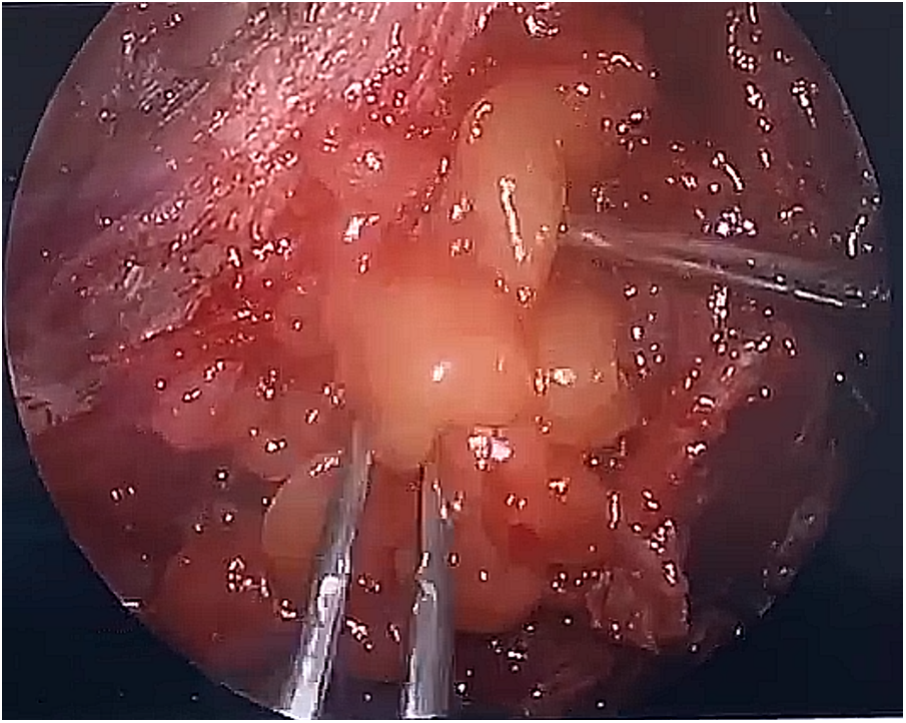
Fat tissue was used to tampon the cystic remnant.

After observing the fistula on the roof of the Tarlov cyst (Figure 6), we closed it with a muscle graft (Figure 7) and filled the cyst cavity with fat tissue (Figure 8). The patient reported relief from back pain on the first day after the surgery, and total pain relief was achieved at the second follow-up. The lumbosacral MR revealed no sign of cyst recurrence (Figure 5B). While the patient experienced partial recovery from urinary incontinence, erectile dysfunction persisted without improvement.

## 4 Discussion

Pathologically, the Tarlov cyst is an abnormal enlargement of the CSF space, which features a ball-valve structure. This unique structure allows CSF to influx from the subarachnoid space while restricting CSF efflux, which keeps Tarlov size promotion. A consensus has been achieved that follow-up should be applied for asymptomatic Tarlov individuals. For symptomatic Tarlov cases, surgical intervention remains the only effective treatment option. However, due to the extensive sacral laminectomies and various unfavorable complications, clinicians are in desperate need of more promising surgical approaches for Tarlov cysts.

Based on the therapeutic underlying logic, the available surgical procedures for Tarlov cysts could be categorized into two categories, which are cyst decompression (either from the cyst inside or outside) and resection. The cystic CSF drainage could decompress the Tarlov cyst from the inside. By inserting a lumboperitoneal shunt into the Tarlov, the cystic hydrostatic/pulsatile pressure decreases significantly, which subsequently lowers the pathological pressure on the nerve root and relieves patients' symptoms (15). However, lumboperitoneal shunt could not be appled to those with multiple nerve root cysts as it is very difficult to identify which cyst shows primary liability for patients' complaints. Additionally, shunt malfunction and infection are also severe complications that prevent this approach from being wildly adopted. Simple decompressive laminectomy is aimed to decompress the cyst from the outside, which however shows a low successful rate due to continuous expansion of the cyst after surgery (16). Cyst resection combined with either neck ligation or cyst wall imbrication demonstrates good outcomes. Nevertheless, due to significant bony loss during cyst resection, the patients may suffer from CSF leakage, nerve root damage, and worsening back pain. Incomplete cyst wall removal could also cause Tarlov cyst recurrence. Thus, neither of the decompression procedures has achieved optimal results so far.

Computed tomography-guided percutaneous cyst drainage (CTG-PCD) is previously reported as a minimally invasive therapeutic for the Tarlov cyst. An early study reported instant relief of sciatica pain after CTG-PCD (8). However, the effect of CTG-PCD remains temporary and cysts usually recur within 3–6 months in most cases. Later, Patel et al. (17) administrated fibrin glue into the Tarlov cysts following CTG-PCD, and all four patients experienced total resolution or marked improvement of symptoms for as long as 23 months. This result was confirmed by several subsequent studies as they achieved similar results (12, 18–20). However the good outcomes, fibrin administration could lead to aseptic meningitis, clinically demonstrated as postprocedural fever and neck stiffness (17). Other complications may also include malaise, headache, nausea, vomiting, and local pain. Although this procedure so far seems to achieve optimal results, the related complications remain a major concern during its clinical usage.

In the current study, we illustrate the technical aspects of the neuro-endoscopic approach in Tarlov cyst management. This procedure not only presents good therapeutic results but also eliminates major complications of previous surgery. Apparent pain relief was observed right after surgery as well as at 3rd month post-surgical period. Additionally, bladder and bowel function recovery were also reported. We believe this good outcome is achieved based on three mechanisms. Firstly, our surgical procedure could effectively decompress the Tarlov cyst. Tarlov patients' symptoms are caused by the pathological pressure on the nerve root from the cyst. Our procedure identifies and seals the communication of the Tarlo cyst. Thus, the CSF from normal subarachnoid space could no longer influx into the Tarlov cyst, which lowers the hydrostatic/pulsatile pressure and thus decreases pathological pressure on the nerve root. Secondly, our approach could effectively prevent the future recurrence of Tarlov. Previously, fibrin administration via CTG-PCD demonstrated a good clinical outcome. We utilize the characteristics of fibrin and ingeniously integrate it with autologous fat to further reinforce the sealing effect on the communication as well as the tamponade of the Tarlov cyst. Thus, double assurance on the leakage point and cyst closure enables us to achieve excellent therapeutic results as presented. Thirdly, the usage of the 30-degree endoscope provides surgeons with a panoramic surgical view, which decreases the possibility of nerve damage and thus lowers the incidence of surgical complications. These features of our procedure enable the good outcomes of Tarlov.

Our study also has some limitations. Firstly, the small sample size and non-blind assessments may generate statistical bias, which requires future large research to confirm the results achieved from the current study. Secondly, although results from short-term follow-ups showed good outcomes, the long-term cure and recurrence rate are required from future follow-ups to verify this result. Thirdly, we did not perform an objective assessment of bladder and anal sphincter structure and function. Instead, a self-reported questionnaire was used to compare the symptoms of improvement regarding incontinence and constipation, which however is common in literature. In our future study, both urodynamics and anorectal manometry will be included to better assess the bladder and bowel function recovery after surgery, respectively. Additionally, although no surgical complication was observed, fever was observed in two patients in our study. Since our surgical procedures require durotomy, bacterial meningitis secondary to cerebrospinal fluid leakage becomes a primary suspect. Another possibility for the fever could be fibrin-related aseptic meningitis (17). Since literature demonstrated that infection is not a major cause of fever after spine surgery (21–23), we suspect that fever in our study could either be surgical-stress-, wound- or fibrin-related. A future study enrolling a large sample size is required to identify the exact cause of fever after neuro-endoscopic assisted surgery.

In addition, we would like to highlight the fact that the results of the current study were obtained from a single-arm study. Thus, the conclusion was drawn based on a paired *t*-test comparison between pre- and post-operative patient data. In the absence of a control arm, precise data on the safety and efficacy of the current surgical technique remain to be determined.

Conclusively, we reported a novel neuro-endoscopic assisted technique to treat Tarlov cysts, which combines the fenestration, leakage seal, and tamponade of the Tarlov cyst. This procedure provides prominent pain and symptom relief while demonstrating no surgical-related complications. Therefore, we believe this procedure could be an effective treatment option for Tarlov cyst.

## Data Availability

The original contributions presented in the study are included in the article/Supplementary Material, further inquiries can be directed to the corresponding authors.
